# Supplementary material for: A highly potent human neutralizing antibody prevents vertical transmission of Rift Valley fever virus in a rat model
Source: Nat Commun. 2023 Jul 26;14:4507. doi: 10.1038/s41467-023-40187-z (PMC10372071; doi:10.1038/s41467-023-40187-z)
Supplement: Supplementary file 1 — Supplementary Information [file 41467_2023_40187_MOESM1_ESM.pdf]

Supplementary data files for:

Title:

A highly potent human neutralizing antibody prevents vertical transmission of Rift Valley fever virus in a rat model

Authors:

Cynthia M McMillen[1,2], Nathaniel S Chapman[3], Ryan M Hoehl[1], Lauren B Skvarca[4], Madeline M Schwarz[1,2], Laura S Handal[5], James E Crowe Jr.*[3,5,6], Amy L Hartman*[1,2]

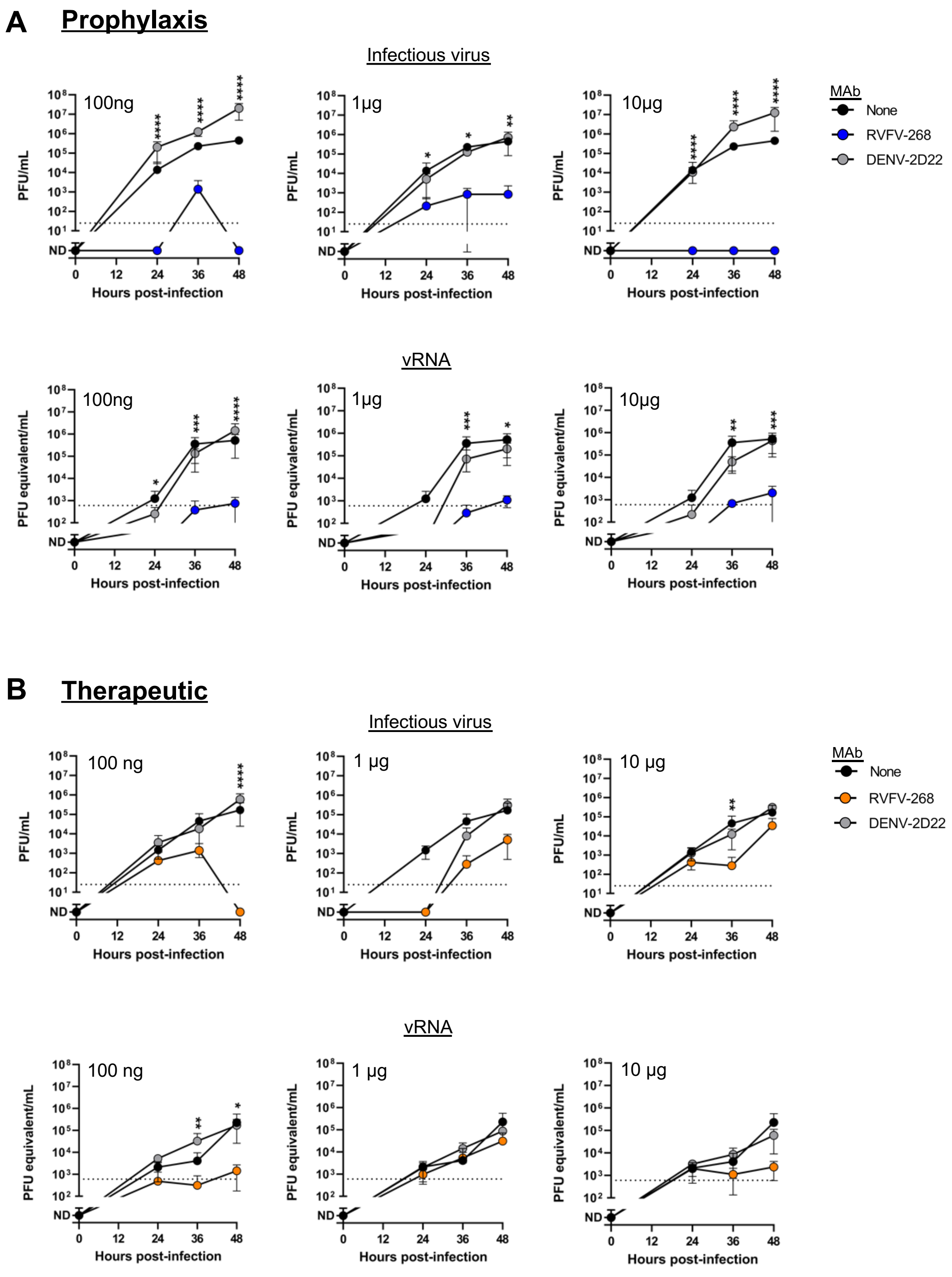

Supplemental Figure 1: MAb RVFV-268 reduces viral titers in rat placenta explant cultures. A) For prophylactic studies, explant slices were incubated with a combination of RVFV (1 x 105 pfu) and mAbs RVFV-268 or DENV-2D22 (100 ng, 1 µg, 10 µg) for 1 hour, then washed and replenished with antibody (100 ng, 1 µg, 10 µg, respectively) in culture media. B) For therapeutic studies, explant slices were incubated with RVFV (1 x 10^5 pfu) for 1 hour, then washed and treated with mAbs RVFV-268 or DENV-2D22 (100 ng, 1 µg, 10 µg) in culture media. Culture supernatant (n=3) was collected at 0, 24, 36, 48 hour time-points then analyzed by VPA (top) or qPCR (bottom). Cultures without antibody (no mAb) served as a positive control. Limit of detection (LOD) = hashed line. Data are presented as mean values +/- standard deviation. Two-sided analysis of variance (ANOVA) was performed.

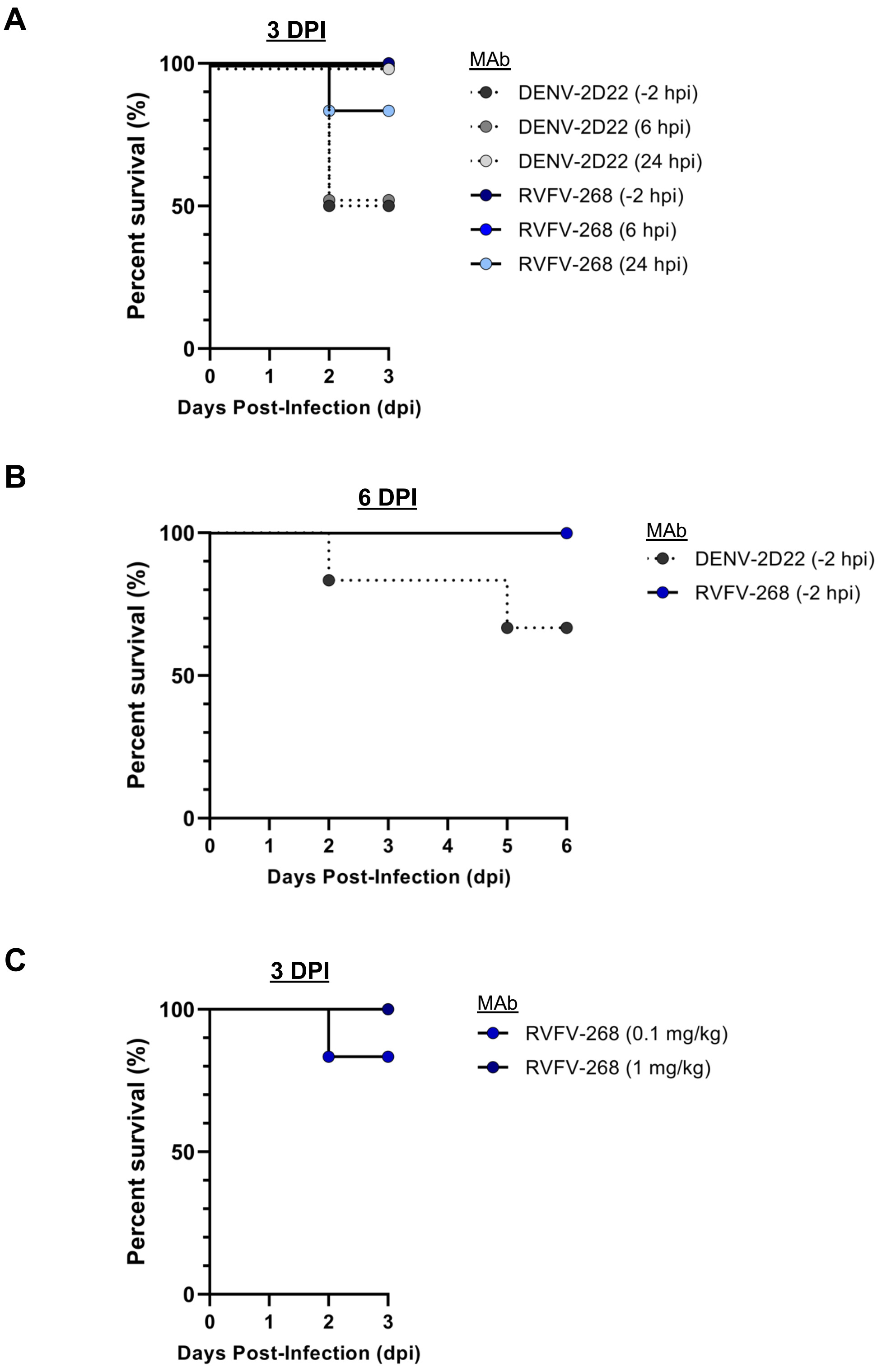

Supplemental Figure 2: Survival curve for dams treated with mAb RVFV-268 or DENV-2D22. Dams were treated with mAbs **A)** at a dose of 10 mg/kg 2 hours prior to RVFV challenge or 6 or 24 hours post RVFV challenge followed by a planned euthanasia at 3 dpi, **B)** at a dose of 10 mg/kg 2 hours prior to RVFV challenge with a planned euthanasia at 6 dpi or **C)** at a dose of 1 mg/kg or 0.1 mg/kg 2 hours prior to challenge with RVFV with euthanasia at 3 dpi.
