# Supplementary material for: A highly potent human neutralizing antibody prevents vertical transmission of Rift Valley fever virus in a rat model
Source: Nat Commun. 2023 Jul 26;14:4507. doi: 10.1038/s41467-023-40187-z (PMC10372071; doi:10.1038/s41467-023-40187-z)
Supplement: Supplementary file 3 — Reporting Summary [file 41467_2023_40187_MOESM3_ESM.pdf]

nature portfolio

Corresponding author(s): James E Crowe, Jr, Amy L Hartman

Last updated by author(s): Jul 11, 2023

Reporting Summary

Nature Portfolio wishes to improve the reproducibility of the work that we publish. This form provides structure for consistency and transparency in reporting. For further information on Nature Portfolio policies, see our Editorial Policies and the Editorial Policy Checklist.

Statistics

For all statistical analyses, confirm that the following items are present in the figure legend, table legend, main text, or Methods section.

n/a	Confirmed	
☐	☒	The exact sample size (*n*) for each experimental group/condition, given as a discrete number and unit of measurement
☒	☐	A statement on whether measurements were taken from distinct samples or whether the same sample was measured repeatedly
☐	☒	The statistical test(s) used AND whether they are one- or two-sided<br>*Only common tests should be described solely by name; describe more complex techniques in the Methods section.*
☐	☒	A description of all covariates tested
☒	☐	A description of any assumptions or corrections, such as tests of normality and adjustment for multiple comparisons
☐	☒	A full description of the statistical parameters including central tendency (e.g. means) or other basic estimates (e.g. regression coefficient) AND variation (e.g. standard deviation) or associated estimates of uncertainty (e.g. confidence intervals)
☐	☒	For null hypothesis testing, the test statistic (e.g. *F*, *t*, *r*) with confidence intervals, effect sizes, degrees of freedom and *P* value noted<br>*Give P values as exact values whenever suitable.*
☒	☐	For Bayesian analysis, information on the choice of priors and Markov chain Monte Carlo settings
☒	☐	For hierarchical and complex designs, identification of the appropriate level for tests and full reporting of outcomes
☒	☐	Estimates of effect sizes (e.g. Cohen's *d*, Pearson's *r*), indicating how they were calculated

Our web collection on statistics for biologists contains articles on many of the points above.

Software and code

Policy information about availability of computer code

Data collection	Excel was used for data collection, calculations, and organization.
Data analysis	GraphPad Prism 9 was used for data analysis and statistics.

For manuscripts utilizing custom algorithms or software that are central to the research but not yet described in published literature, software must be made available to editors and reviewers. We strongly encourage code deposition in a community repository (e.g. GitHub). See the Nature Portfolio guidelines for submitting code & software for further information.

Data

Policy information about availability of data

All manuscripts must include a data availability statement. This statement should provide the following information, where applicable:

- Accession codes, unique identifiers, or web links for publicly available datasets
- A description of any restrictions on data availability
- For clinical datasets or third party data, please ensure that the statement adheres to our policy

All data needed to evaluate the conclusions in this manuscript are present in the manuscript and/or the Supplementary Materials. The data generated in this study was stored in Microsoft Excel for Microsoft 365 MSO (Version 2306 Build 16.0.16529.20100) and graphed using GraphPad Prism v9.

Human research participants

Policy information about studies involving human research participants and Sex and Gender in Research.

Reporting on sex and gender	n/a
Population characteristics	n/a
Recruitment	n/a
Ethics oversight	n/a

Note that full information on the approval of the study protocol must also be provided in the manuscript.

Field-specific reporting

Please select the one below that is the best fit for your research. If you are not sure, read the appropriate sections before making your selection.

☒ Life sciences ☐ Behavioural & social sciences ☐ Ecological, evolutionary & environmental sciences

For a reference copy of the document with all sections, see nature.com/documents/nr-reporting-summary-flat.pdf

Life sciences study design

All studies must disclose on these points even when the disclosure is negative.

Sample size	Sample sizes were determined based on the numbers used in previous studies that resulted in sufficient data to perform statistical analyses. For each test cohort we include 5-6 rats, however once we observed consistent vertical transmission of RVFV in DENV-2D22 mAb treated groups, we down selected to 1 rat per experiment (2 total rats per research question) to reduce animal consumption.<br><br>References:<br>McMillen, C. M. et al. Rift Valley fever virus induces fetal demise in Sprague-Dawley rats through direct placental infection. Sci Adv 4, eaau9812, doi:10.1126/sciadv.aau9812 [doi] aau9812 [pii] (2018).<br>McMillen, C. M. et al. Congenital Rift Valley fever in Sprague Dawley rats is associated with diffuse infection and pathology of the placenta. PLoS neglected tropical diseases 16, e0010898, doi:10.1371/journal.pntd.0010898 (2022).
Data exclusions	Data from uninfected controls was not included in the study given the null results. Furthermore, ELISA data from DENV-2D22 animals was not included in the results given the null results. Negative values from these controls were expected and inclusion of the data in the manuscript would distract from the main conclusions of the study.
Replication	For therapeutic and prophylactic treatment studies in vivo studies at least 2 experiments were performed with multiple (n=2 or 3) biological replicates per treatment group, with the exception of reducing the number of DENV-2D22 control mAb treated animals to 1 from 2 per experiment to reduce animal consumption. This reduction was only performed after we confirmed that dam infection and vertical transmission of RVFV was reproducible in the mAb DENV-2D22 treated animals 100%. Our results are reproducible.
Randomization	Animals were randomly placed into treatment groups. For placenta explant studies, similar sized placenta sections were selected and placed evenly across treatment groups to prevent bias.
Blinding	Protocol and identification development, animal treatments, euthanasia, sample processing, sample analysis was performed by the same individuals, therefore ELISA, qPCR, and VPA analyses were not blinded. Microscopic histopathologic examination was performed by a board-certified anatomic pathologist, blinded to treatment groups

Reporting for specific materials, systems and methods

We require information from authors about some types of materials, experimental systems and methods used in many studies. Here, indicate whether each material, system or method listed is relevant to your study. If you are not sure if a list item applies to your research, read the appropriate section before selecting a response.

Materials & experimental systems

n/a	Involved in the study	
[ ]	[x]	Antibodies
[ ]	[x]	Eukaryotic cell lines
[x]	[ ]	Palaeontology and archaeology
[ ]	[x]	Animals and other organisms
[x]	[ ]	Clinical data
[x]	[ ]	Dual use research of concern

Methods

n/a	Involved in the study	
[x]	[ ]	ChIP-seq
[x]	[ ]	Flow cytometry
[x]	[ ]	MRI-based neuroimaging

Antibodies

Antibodies used

1)A custom rabbit anti-RVFV nucleoprotein polyclonal antibody was generated by Genscript.
2) goat anti-rabbit IgG, labeled with Alexa Fluor-594 (Invitrogen A11012)
3) mAbs RVFV-268 and DENV-2D22 generated in-house by the Crowe Lab
4) goat-anti-human-HRP conjugated IgG [200 ng/mL; 1:5,000 dilution] (Invitrogen; 62-8420)

Validation

1) Custom rabbit anti-RVFV nucleoprotein pAb -
Details of PolyExpress™ Custom Polyclonal Antibody Production Services

Details from Genscript website::

Feature Details SC1180 SC1676
Immunization Antigen Synthesized 1 peptide 1 protein
No. of rabbits immunized 2
Deliverables Negative Control Total IgG (optional: Pre-immune serum or total serum protein delivery)
Antigen Sample 2 mg of Peptide Antigen 200 µg Protein
Antigen Affinity Purified pAb(s) 1 (2-6 mg total) 2 (3-12 mg total)
Guarantee Application ELISA titer 1:256,000 WB positive for target protein antigen binding; ELISA Titer 1:256,000
Antibody Yield 1 purified pAb ≥ 2 mg 2 separately purified pAbs (1.5 – 6mg/ rabbit)
Timeline 45 days 10-11 weeks
*GenScript provides peptide synthesis, peptide design, carrier conjugation and antibody purification for free.
**During the development of the antibody, if there is the death of the experimental animal, we will choose the adjusted immunization process for re-immunization to ensure the high quality and efficiency of the order.

2) Goat anti-rabbit IgG, labeled with Alexa Fluor-594 (Invitrogen A11012) -
Product Specific Information from Invitrogen website:
To minimize cross-reactivity, these goat anti-rabbit IgG whole antibodies have been cross-adsorbed against human IgG, human serum, mouse IgG, mouse serum, and bovine serum. Cross-adsorption or pre-adsorption is a purification step to increase specificity of the antibody resulting in higher sensitivity and less background staining. The secondary antibody solution is passed through a column matrix containing immobilized serum proteins from potentially cross-reactive species. Only the nonspecific-binding secondary antibodies are captured in the column, and the highly specific secondaries flow through. The benefits of this extra step are apparent in multiplexing/multicolor-staining experiments (e.g., flow cytometry) where there is potential cross-reactivity with other primary antibodies or in tissue/cell fluorescent staining experiments where there are may be the presence of endogenous immunoglobulins.

Alexa Fluor dyes are among the most trusted fluorescent dyes available today. Invitrogen™ Alexa Fluor 594 dye is a bright, red-fluorescent dye with excitation ideally suited to the 594 nm laser line. For stable signal generation in imaging and flow cytometry, Alexa Fluor 594 dye is pH-insensitive over a wide molar range. Probes with high fluorescence quantum yield and high photostability allow detection of low-abundance biological structures with great sensitivity. Alexa Fluor 594 dye molecules can be attached to proteins at high molar ratios without significant self-quenching, enabling brighter conjugates and more sensitive detection. The degree of labeling for each conjugate is typically 2-8 fluorophore molecules per IgG molecule; the exact degree of labeling is indicated on the certificate of analysis for each product lot.

Using conjugate solutions: Centrifuge the protein conjugate solution briefly in a microcentrifuge before use; add only the supernatant to the experiment. This step will help eliminate any protein aggregates that may have formed during storage, thereby reducing nonspecific background staining. Because staining protocols vary with application, the appropriate dilution of antibody should be determined empirically. For the fluorophore-labeled antibodies a final concentration of 1-10 µg/mL should be satisfactory for most immunohistochemistry and flow cytometry applications.

Product will be shipped at Room Temperature.

Target Information
Anti-Rabbit secondary antibodies are affinity-purified antibodies with well-characterized specificity for rabbit immunoglobulins and are useful in the detection, sorting or purification of its specified target. Secondary antibodies offer increased versatility enabling users to use many detection systems (e.g. HRP, AP, fluorescence). They can also provide greater sensitivity through signal amplification as multiple secondary antibodies can bind to a single primary antibody. Most commonly, secondary antibodies are

generated by immunizing the host animal with a pooled population of immunoglobulins from the target species and can be further purified and modified (i.e. immunoaffinity chromatography, antibody fragmentation, label conjugation, etc.) to generate highly specific reagents.

3) mAbs RVFV-268 and DENV-2D22:
The human monoclonal antibodies that are the focus of this manuscript are novel clones isolated from human individuals with laboratory-confirmed previous infection. The antibody specificities were validated by 1) binding assays using recombinant antigens and cell surface displayed antigens, and 2) neutralization of authentic viruses.

4) goat-anti-human-HRP conjugated IgG [200 ng/mL; 1:5,000 dilution] (Invitrogen; 62-8420). References and validation of this antibody is provided on the following datasheet on the Invitrogen website. (https://www.thermofisher.com/order/genome-database/dataSheetPdf?producttype=antibody&productsubtype=antibody_secondary&productId=62-8420&version=322)

Eukaryotic cell lines

Policy information about cell lines and Sex and Gender in Research

Cell line source(s)	Vero E6 cells (ATCC:CRL-1586)
Authentication	Vero E6 cells were not authenticated other than the authentication that is provided by the company that propagates the cells (ATCC).
Mycoplasma contamination	Vero E6 cells were spot-checked for mycoplasma contamination. Cells used in this study were negative for mycroplasma contamination.
Commonly misidentified lines (See ICLAC register)	No commonly misidentified cell lines were used in this study.

Animals and other research organisms

Policy information about studies involving animals; ARRIVE guidelines recommended for reporting animal research, and Sex and Gender in Research

Laboratory animals	Sprague Dawley Rats, 6-8 weeks old
Wild animals	This study did not involve wild animals.
Reporting on sex	Sex differences were not analyzed in this study given that it focuses on the prevention of vertical transmission of RVFV with monoclonal antibodies. Only pregnant female rats were pertinent to the study.
Field-collected samples	The study did not involve samples collected from the field.
Ethics oversight	All animal work described in this study was carried out in strict accordance with the Guide for the Care and Use of Laboratory Animals of the NIH and the Animal Welfare Act. The protocol was approved and overseen by the University of Pittsburgh Institutional Animal Care and Use Committee. The Association for Assessment and Accreditation of Laboratory Animal Care has fully accredited the University of Pittsburgh.

Note that full information on the approval of the study protocol must also be provided in the manuscript.
